# Genomic microsatellite characteristics analysis of *Dysommaanguillare* (Anguilliformes, Dysommidae), based on high-throughput sequencing technology

**DOI:** 10.3897/BDJ.11.e100068

**Published:** 2023-04-07

**Authors:** Ziyan Zhu, Yuping Liu, Shufei Zhang, Sige Wang, Tianyan Yang

**Affiliations:** 1 Zhejiang Ocean University, Zhoushan, China Zhejiang Ocean University Zhoushan China; 2 Guangdong Provincial Key Laboratory of Fishery Ecology and Environment, South China Sea Fisheries Research Institute, Guangzhou, China Guangdong Provincial Key Laboratory of Fishery Ecology and Environment, South China Sea Fisheries Research Institute Guangzhou China

**Keywords:** *Dysommaanguillare*, genome, microstatellite, high-throughput sequencing

## Abstract

Microsatellite loci were screened from the genomic data of *Dysommaanguillare* and their composition and distribution were analysed by bioinformatics for the first time. The results showed that 4,060,742 scaffolds with a total length of 1,562 Mb were obtained by high-throughput sequencing and 1,160,104 microsatellite loci were obtained by MISA screening, which were distributed on 770,294 scaffolds. The occurrence frequency and relative abundance were 28.57% and 743/Mb, respectively. Amongst the six complete microsatellite types, dinucleotide repeats accounted for the largest proportion (592,234, 51.05%), the highest occurrence frequency (14.58%) and the largest relative abundance (379.27/Mb). A total of 1488 microsatellite repeats were detected in the genome of *D.anguillare*, amongst which the hexanucleotide repeat motifs were the most abundant (608), followed by pentanucleotide repeat motifs (574), tetranucleotide repeat motifs (232), trinucleotide repeat motifs (59), dinucleotide repeat motifs (11) and mononucleotide repeat motifs (4). The abundance of microsatellites of the same repeat type decreased with the increase of copy numbers. Amongst the six types of nucleotide repeats, the preponderance of repeated motifs are A (191,390, 43.77%), CA (150,240, 25.37%), AAT (13,168, 14.05%), CACG (2,649, 8.14%), TAATG (119, 19.16%) and CCCTAA (190, 19.16%, 7.65%), respectively. The data of the number, distribution and abundance of different types of microsatellites in the genome of *D.anguillare* were obtained in this study, which would lay a foundation for the development of high-quality microsatellite markers of *D.anguillare* in the future.

## Introduction

Shortbelly eel (*Dysommaanguillare* Barnard, 1923) is a small-sized warm water eel that is widely distributed in the Indian Ocean and the western Pacific Ocean (Nelson et al. 2016). In China, it is also one of the preponderant bycatch in the offshore waters of the southern East China Sea (Zhao et al. 2016). As an intermediate to high trophic-level species in the coastal food webs, it is of great significance in the offshore marine ecosystem and biodiversity. However, the limited studies of *D.anguillare* were mainly focused on the nutrition and feeding habits (Zhang and Tang 2003), the spatial-temporal pattern of community structure (Liu and Xian 2009) and the effects of lipid removal on the stable isotopes (Yang et al. 2020).

The explicit germplasm genetic characteristics of fishery species are considered to be the indispensable prerequisite for effective fisheries management (Hemmer-Hansen et al. 2018). However, the available genetic data for this species are still scarce and only partial mitochondrial and nuclear gene sequences have hitherto been reported and analysed (Chen et al. 2014, Chang et al. 2016, Wang et al. 2019). Microsatellite DNA, also named simple sequence repeats (SSRs) are short tandem duplications (typically 1-6 nucleotide repeats and mostly less than 100 bp in length), ubiquitous occurring in eukaryotic organisms. Besides, the repetitions vary drastically amongst different genotype of the same species (Tautz and Renz 1984). The co-dominant microsatellite molecular markers, based on polymerase chain reaction (PCR) techniques, have overriding advantages in high polymorphism, good repeatability, simple operation and low experimental cost. Therefore, it has possessed important applied worth in gene mapping and QTL analysis, population genetics and evolutionary research, as well as molecular marker-assisted breeding (Messier et al. 1996, Schlötterer 2000). At present, the conventional development strategies of representative microsatellite loci mainly include anchored-PCR-based method, selective hybridisation enrichment method, database search and relative species selection method (Sun et al. 2009). Nevertheless, these above-mentioned technical means not only are time-consuming and expensive, but also reflect incomplete distribution of microsatellites and develop limited molecular markers.

In recent years, along with the rapid progress of high-throughput sequencing (HTS) technology and the reduction of sequencing cost, developing numerous high-polymorphism SSR markers from multi-omics data has become more and more convenient. In this study, the genome-wide sequences of *Dysommaanguillare* were obtained, based on HiSeq^TM^ 4500 platform for the first time; meanwhile, the SSR loci distribution and characteristics were also analysed by bioinformatics tools. The findings will help to provide useful references and basic information for germplasm resources conservation, population genetic evaluation and phylogenetic relationships analysis amongst related species of Anguilliformes.

## Material and Methods

### Sample collection and genomic DNA extraction

Fifty-three samples of *Dysommaanguillare* were collected by trawling in the coastal waters of Zhoushan, Zhejiang Province in September 2022. After preliminary morphological identification, muscle tissues from five male and five female individuals were randomly selected for the genomic DNA extraction by the traditional Tris-saturated phenol method (Maniatis et al. 1982). Subsequently, the DNA barcode method, based on the mitochondrial COI sequence, was further conducted to ensure the species accuracy . The 1% agarose-gel electrophoresis and NanoDrop 2000 ultraviolet spectrophotometer (USA, Thermo Fisher Scientific) were performed to detect the integrity and purity of the genomic DNA, respectively. The obtained DNA samples were stored at -20℃ for further analysis.

### Library construction and high-throughput sequencing

Equal amounts of DNA (2 μg each) were mixed for library construction and next-generation sequencing by Onemore Technology (Wuhan) Co., Ltd. The genomic DNA was randomly fragmented using Covaris Ultrasonic Processor into small 200 to 350 bp fragments. Two pair-end DNA libraries were constructed through terminal repair, adding Poly-A tails and sequencing adapters, purification and PCR amplification and then sequenced using the Illumina HiSeq^TM^ 4500 sequencing technology.

### Sequence cleaning and genome assembly

Raw data output from Illumina platform were firstly transformed into sequence reads by base calling and recorded in a FASTQ format. Subsequently, clean reads were obtained after filtering adaptor sequences and low quality read by Cutadapt v.1.16 (Martin 2011). SOAPdenovo v.2.04 was used to assemble the clean data with the setting parameters “-K 53 -R -M 3 -d 1”, which employed the *de Bruijn* graph-based assembly strategy (Kajitani et al. 2014). First, reads sequenced from the small-fragment library were divided into smaller substrings (*K-mers*) to construct a preliminary *de Bruijn* diagram. Then, the simplified *de Bruijn* graph was obtained after removing the low-coverage branches and branches that cannot be connected further due to sequencing errors and the sequences at every bifurcation locus were truncated to obtain the initial contigs. By mapping the paired-end reads back to the contigs, the connectivity relationships between the reads and the information of the inserted fragment size were used to further assemble the contigs into scaffolds and obtain the primary genomic sequence.

### Screening and identification of SSRs

MicroSatellite identification tool (MISA) software (http://pgrc.ipk-gatersleben.de/misa/) written by Perl script was implemented to scan the assembled scaffolds to identify the genome-wide microsatellite repeat units and to analyse the length, location and quantity of the SSRs (Thiel et al. 2003). The occurrence frequency of SSR loci, average distribution distance and density of microsatellites, type and length of repeat motifs were calculated using Microsoft Excel 2019. The default parameters of MISA were set as follows: the repeat motif length was from 1 to 6 nucleotides and the minimum thresholds of repeat counts were 1-10, 2-6, 3-5, 4-5, 5-5 and 6-5, which meant the number of mononucleotide repeats was less than 10, number of dinucleotide repeats was less than 6 and numbers of remaining repeats were all less than 5, respectively. Besides, the number of bases interrupting two SSRs in a compound microsatellite should be less than 100. Considering the Watson-Crick complementary condition and the difference in the base arrangement, the repeat sequences and their complementary sequences were grouped together. For example, the (AC)_n_, (CA)_n_, (TG)_n_ and (GT)_n_ were treated as the same SSR repeat types.

## Results

### Genome sequencing and assembly

The information of contigs and scaffolds of the *Dysommaanguillare* genome was listed in the Table 1. About 11,805,379 contigs with the total length 1,960 Mb were obtained after splicing and the average GC content was about 42.2%. The number of scaffolds produced by the SOAPdenovo v.2.0 assembly was 4,060,742 and the full length was 1,561 Mb, with the average GC content 39.6%.

N50 value is a widely used metric for measuring the quality of sequences by the assembly algorithms' output. It refers to the contig or scaffold length value when the accumulated fragment length (from long to short) exceeds 50% of the total length of all contigs or scaffolds for the first time. The greater the N50 value, the smaller the quantity and the better the assembly quality. In this study, the N50 values of contig and scaffold assembly were 272 bp and 709 bp, respectively. Compared with the assembled genomes of related species *Anguillajaponica* (*Henkel et al. 2012*), *A.anguilla* (Jansen et al. 2017) and *A.rostrate* (Pavey et al. 2017), the assembly effect of *Dysommaanguillare* was relatively good and developing microsatellite markers could reflect the genome-wide characteristics of SSRs.

### SSR repeat types and distribution

A total of 1,160,104 microsatellites with 1-6 bp nucleotide motifs were detected in 770,294 unigenes and 234,959 of them contained more than one SSR locus, with the occurrence frequency (total number of SSRs detected/total number of unigenes) of 28.57%. The density of distribution (total length of unigenes/total number of SSRs screened) was on average 1/1.35 kb and the relative abundance (total number of SSRs screened/total length of unigenes) was 743/Mb.

**Figure 1.**
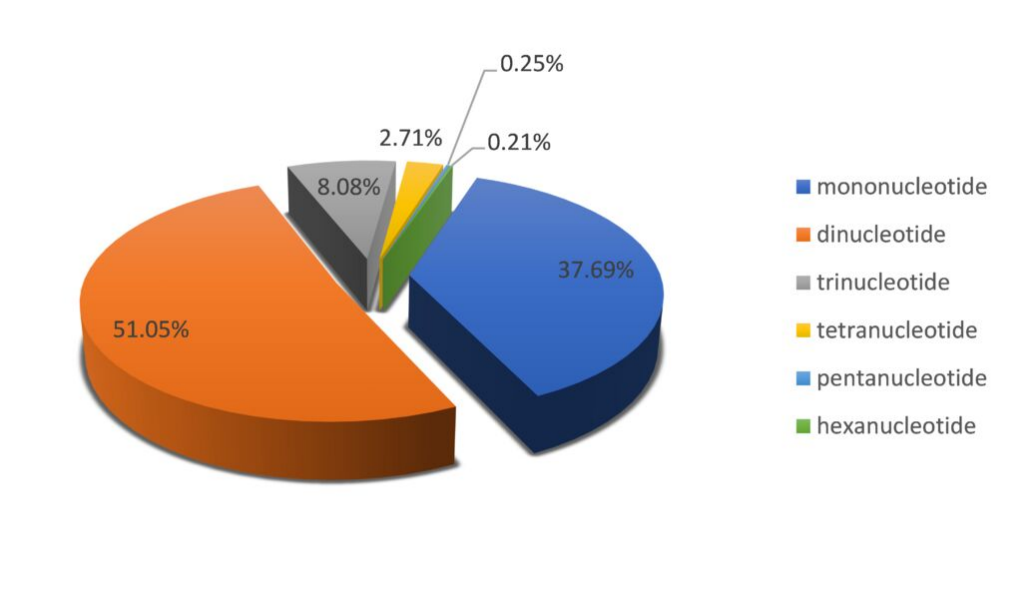
Distribution of SSRs repeat types in genomes of *D.anguillare*.

These SSR loci can be classified into six repeat types: mononucleotide, dinucleotide, trinucleotide, tertranucleotide, pentanucleotide and hexanucleotide. The most abundant type of repeat motif was dinucleotide, accounting for 51.05% in the all SSR loci and then followed by mononucleotide (37.69%), trinucleotide (8.08%), tertranucleotide (2.71%) and pentanucleotide (0.25%), while hexanucleotide was the minimum (0.21%) of all (Fig. 1). The occurrence frequency of dinucleotide repeats was highest, while hexanucleotide was observed the lowest, representing 14.58% and 0.06% of the total genome, respectively. The relative abundance of dinucleotide reached 379.27/Mb, with an average of one SSR locus per 2.64 kb and the next was mononcleotide (280.00/Mb). By comparison, the relative abundance of hexanucleotide was the lowest (1.59/Mb) (Table 2).

### Repeat numbers of different SSRs

**Figure 2.**
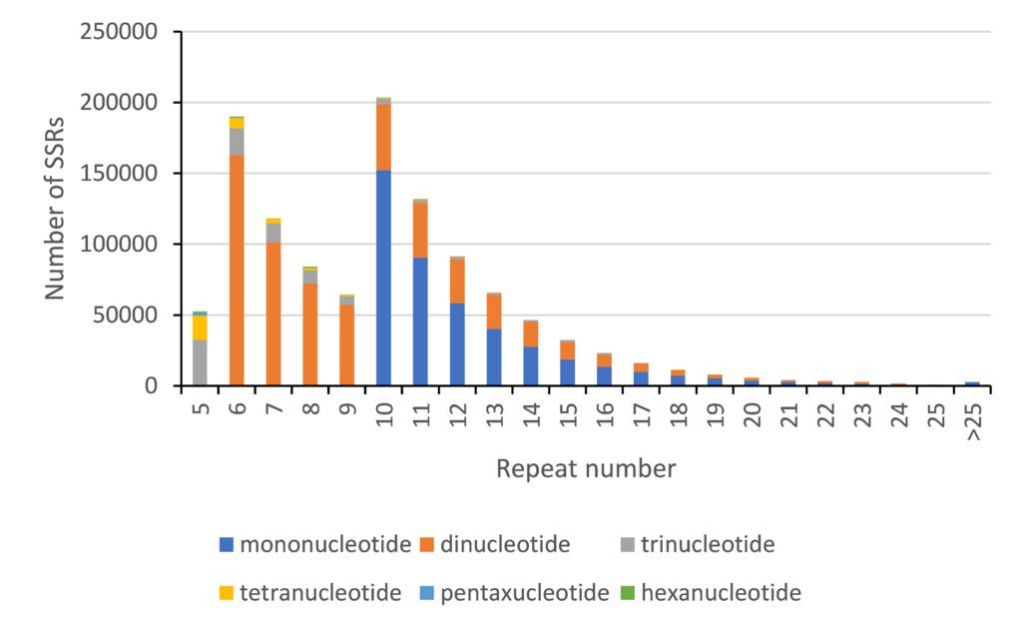
SSR repeats distribution of *D.anguillare*.

The number of repeats of SSR loci mainly ranged from 5 to 24. The predominant repeat number of the SSR loci was 10 times, comprising 17.52% of the total number of SSR loci. In general, the number of repeat types decreased with the increase in repeat numbers (Fig. 2). The repeats of mononucleotide, dinucleotide and trinucleotide were mainly distributed in 10-19 times (96.83%), 6-15 times (95.15%) and 5-9 times (85.34%), respectively. However, the repeat times of the rest of the repeat types were all within 13 times, which were mainly in the range of 5-8 times and separately accounted for 92.40%, 96.70% and 99.56% (Table 3).

In summary, the repeat numbers of SSR loci were mainly concentrated in 10-15 times and 5-8 times, with a total number of 1,016,359 (87.61%). Few SSR loci with more than 25 repeats were identified and the type of base repeats was monotonous, only composing of mononucleotide repeat.

### Copy numbers of repeat units

**Figure 3.**
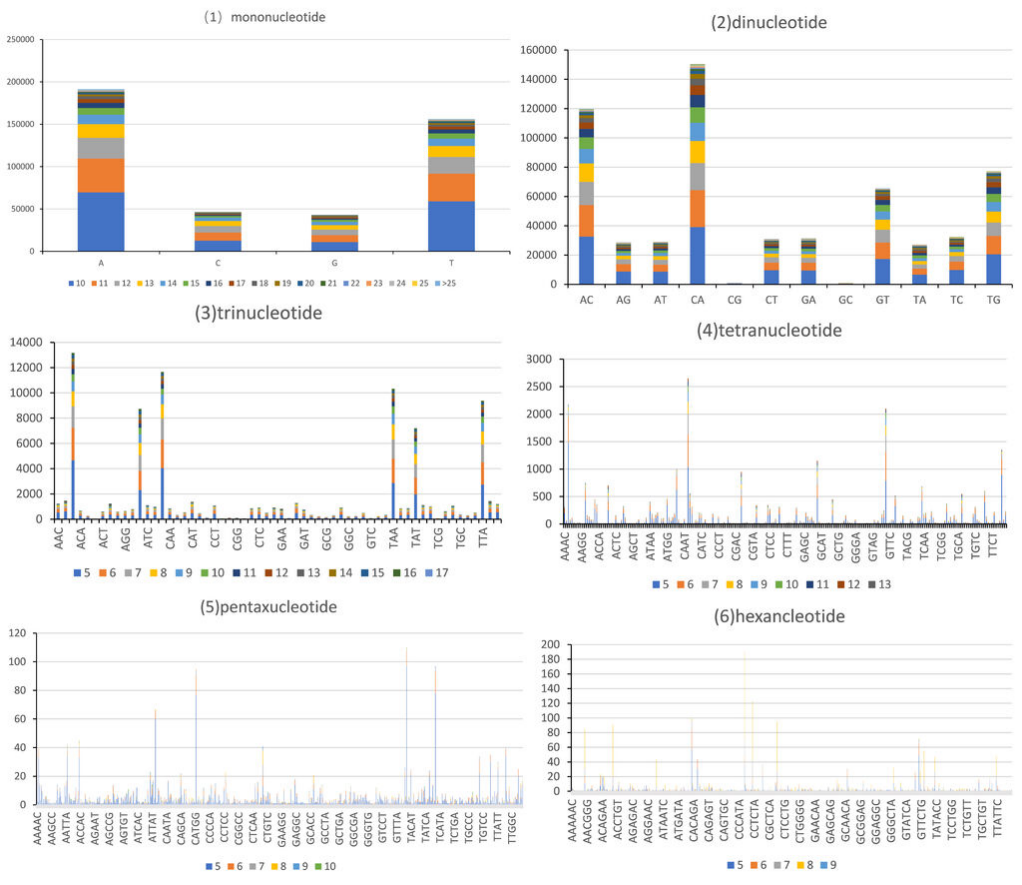
The distribution of microsatellite repeats in genome of *D.anguillare*.

Amongst the detected 1,488 repeat units, hexanucleotide repeats possessed the most types and pentanucleotide repeats took second place. Nevertheless, the type of mononucleotide repeats was the least limited to the base number (Table 4). Amongst all these repeats, the dominant repeat motifs in mononucleotide, dinucleotide, trinucleotide, tetranucleotide, pentanucleotide and hexanucleotide were A (191,390, 43.77%), CA (150,240, 25.37%), AAT (13,168, 14.05%), CACG (2,649, 8.14%), TAATG (119, 19.16%) and CCCTAA (190, 7.65%), respectively (Fig. 3, Table 4).

### SSR length distribution and polymorphism evaluation

**Figure 4.**
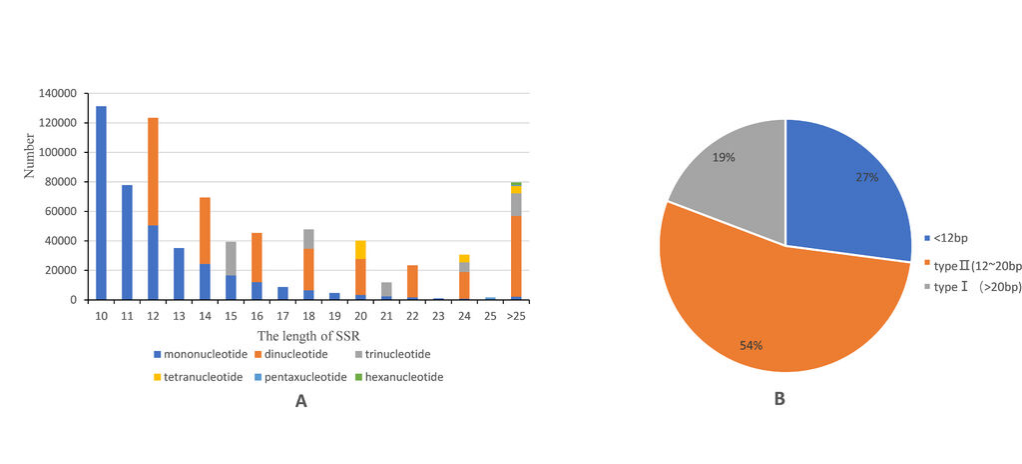
Length distribution of genes in *D.anguillare*. **A** SSR length distribution; **B** Distribution types of SSR (type I and type II).

The sequence length amongst different types of SSRs varied a lot, from 10 to 54 bp (Fig. 4). The minimum and maximum variations in length were detected in hexanucleotide and mononucleotide repeats, respectively. The former was in the range of 30-54 bp with the total length of 1,774 bp, while the latter was in the range of 10-51 bp with total length of 379,455 bp, which constituted approximately 49.14% of the total length of SSRs. Amongst the six types of nucleotide repeat, dinucleotide and trinucleotide were dominant in the distribution of microsatellites from the perspective of sequence length, which were 677,805 bp in total and accounting for 87.78% in all SSRs.

The length of the microsatellite was one of the main factors affecting its polymorphism. Temnykh et al. (2001) divided SSR sequences into two categories: the high-polymorphic type I (length ≥ 20 bp) and the moderate-polymorphic type Ⅱ (12 bp ≤ length < 20 bp). The microsatellites with length less than 12 bp owned lower polymorphism, but higher mutation potential. In the present study, there were 21,347 type I SSRs (19%) and 294,373 type II SSRs (54%), respectively. SSR loci with low mutation potential accounted for 27%.

## Discussion

### Number and relative abundance of microsatellites in the genome of Dysommaanguillare

The bioinformatics software was used to search and analyse the various types and numbers of six perfect microsatellites in the genome of *Dysommaanguillare*. Approximately 1,160,104 microsatellite loci were revealed across the 1.56 Gb genome sequence, with a total length of 24,707,980 bp (occupying 58% of the full genome length). In contrast to other published genomes of bony fishes, it was higher than *Takifugurubripes* (0.77%) (Cui et al. 2006), *Scleropagesformosus* (0.78%) (Duan et al. 2019) and *Bagariusyarrelli* (1.23%) (Yang et al. 2021), but lower than *Pelteobagrusfulvidraco* (1.80%) (Xu et al. 2020) and *Harpadonnehereus* (2.01%) (Yang et al. 2021), indicating that genome-wide microsatellites content was not directly related to the genetic relationship and the reasons might involve different retrieval tools, parameter settings and databases (He et al. 2015). Hancock (1996) speculated that the numbers of microsatellites increased with the chromosome length and the disproportional relationship between the genome size and microsatellite numbers was also confirmed in our study.

Relative abundance was an important feature to measure microsatellite richness. It was calculated to be 743/Mb of *Dysommaanguillare*, which was much higher than that of other marine fishes, such as *Scatophagusargus* (653/Mb) (Wang et al. 2020), *Cociellacrocodilus* (428/Mb) (Zhao et al. 2021), *Tridentigerbifasciatus* (347/Mb) (Zhao et al. 2022) and four species of pufferfishes (365/Mb in *Takifugurubripes*, 369/Mb in *Takifuguflavidus*, 397/Mb in *Takifugubimaculatus* and 525/Mb in *Tetraodonnigroviridis*) (Xu et al. 2021). The above result showed that abundant microsatellites existed in the genome of *D.anguillare*, which would provide sufficient molecular markers for the further germplasm identification and genetic diversity studies.

### Distribution characteristics of microsatellites in the genome of Dysommaanguillare

Varied microsatellite types composing of 1-6 nucleotide repeats were discovered in the genome of *Dysommaanguillare* and dinucleotide repeats were the most frequent, followed by mononucleotide repeats, while the percentages of SSRs containing 3-6 nucleotide repeats were no more than 10%. Therefore, priority should be given to dinucleotide repeats when designing SSR primers of *D.anguillare*. Mononucleotide and dinucleotide repeats were regarded as the most abundant types of SSRs in most species. It was reported that mononucleotide repeats tended to dominate in the genomes of higher grade organisms (Gao and Kong 2005). However, dinucleotide repeats contained higher proportions in fish genomes, which probably related to the differences in gene expression and regulation.

The CA repeat motif was the most abundant amongst dinucleotide repeats and occupied 25.37% of them, which was consistent with *Scophthalmusmaximus* (Ruan 2009) and pufferfishes (Cui et al. 2006, Xu et al. 2021), but different from *Ictaluruspunctatus* (Tang et al. 2022), while the number of GC repeat motifs was the least. The base sliding might generate microsatellites more easily at the low melting temperature (T_m_). Two hydrogen bonds between A-T base pairs were more likely to be broken than three hydrogen bonds between G-C base pairs, resulting in reduction of the GC repeats (Huang et al. 2020). Some other scholars pointed out that the methylation of CpG might cause the spontaneous deamination of cytosine to thymine in order to maintain the thermodynamic stability of the DNA molecule. In this study, the proportion of GC repeats motif was only 0.1% and from this aspect, the lower GC content in the whole genome also reflected the small amount of GC repeats (Schorderet and Gartler 1992).

The structural instability and composition of trinucleotide repeats were closely related to some genetic diseases in humans (Sinden et al. 2002). It was found that AAT repeat motif was the most numerous of the trinucleotide repeats in the *Dysommaanguillare* genome, the same as for humans and primates (Kelkar et al. 2008). Therefore, in-depth analysis of trinucleotide repeats would contribute to predict some gene loci associated with human diseases and thereby reduces the occurrence of certain illness by changing gene expression.

### Copy numbers and length variations in the genome of Dysommaanguillare

The repeat unit length was in inverse proportion to the copy number of microsatellite DNA (Harr and Schlötterer 2000). Commonly, the higher the copy number of SSRs meant the more alleles and the richer polymorphism. The number of microsatellite repeats in the *Dysommaanguillare* genome was mainly in the range of 5 to 25. Motifs that showed more than 25 reiterations were very rare (only 2,712 SSRs) and all of them were composed of mononucleotide repeats. Previous studies proved that the mutation rate of microsatellites was positively correlated to the copy number of the repeat motif (Wierdl et al. 1997) and longer microsatellites were expected to have higher mutation rate owing to more chances of replication slippage (Calabrese and Sainudiin 2005). The results demonstrated that the number of SSRs decreased as the repeat number increased. In addition, tetranucleotide, pentanucleotide and hexanucleotide microsatellites might have higher mutation rates than those of the mononucleotide, dinucleotide and trinucleotide microsatellites.

The length of microsatellites in the *Dysommaanguillare* genome was generally 10-18 bp and the number of microsatellites was inversely proportional to the repeat motif length. The structure and its characteristics analysis of a parthenogenic gastropod *Melanoidestuberculata* concluded that the longer the repeat sequence length was, the greater the selection pressure undergoing and the lower numbers of repeats was (Samadi et al. 1998). This phenomenon had been verified by various kinds of plants and animals, for instance, *Juglansregia* (Liao et al. 2014), *Patinopectenyessoensis* (Ni et al. 2018) and *Phrynocephalusaxillaris* (Song et al. 2019). According to Temnykh et al. (2001), SSR polymorphism could be considered low, medium and high and the SSRs with lengths longer than 12 bp were potential molecular markers with high polymorphism. In the study, the type I and type II SSRs in the *D.anguillare* genome occupied about 73% of the total, showing great potential for polymorphism microsatellite development.

## Conclusions

In conclusion, MISA software was used for the first time to search and analyse six types of perfect microsatellite loci from the whole genome survey data of *Dysommaanguillare*. The results showed that both the relative abundance and density of various microsatellite types were very high. Amongst the 1,160,104 SSR loci, the number of different repeat types presented a trend as: dinucleotide > mononucleotide > trinucleotide > tetranucleotide > pentanucleotide > hexanucleotide. The dominant repeat motifs of them were A, CA, AAT, CACG, TAATG and CCCTAA, respectively. The results supplemented the genetic marker database of marine fishes and provided valuable information resources for further genetic analysis of *D.anguillare*.

## Figures and Tables

**Table 1.** The contig and scaffold assembly results statistics.

Assembly level	The total length (bp)	The sequence number	Length number of sequences ≥ 2Kb	The maximum length (bp)	N50 (bp)	N90 (bp)	GC content (%)
Contig	1,960,673,378	11,805,379	30,667	9,646	272	60	42.2
Scaffold	1,561,530,495	4,060,742	95,727	23,878	709	134	39.6

**Table 2.** Proportions of each SSR repeat types in the genome of *D.anguillare*.

Repeat type	Number	Occurrence frequency (%)	Relative abundance (per・Mb ^-1^)	Average length (bp)	Total length (bp)
Mononucleotide	437,234	10.77%	280.00	0.87	379,455
Dinucleotide	592,234	14.58%	379.27	0.50	298,350
Trinucleotide	93,734	2.31%	60.03	0.72	67,533
Tetranucleotide	31,481	0.78%	20.16	0.72	22,680
Pentanucleotide	2,936	0.07%	1.88	0.82	2,409
Hexanu cleotide	2,485	0.06%	1.59	0.71	1,774
Total	1,160,104	28.57%	742.93	4.35	772,201

**Table 3.** Distribution interval of the copy number in different microsatellite motif for *D.anguillare*.

Repeat number	Mononu cleotide	Dinu cleotide	Trinu cleotide	Tetranu cleotide	Pentanu cleotide	Hexanu cleotide	Total	Proportion (%)
5	0	0	32,413	17,143	2,071	972	52,599	4.53%
6	0	162,916	18,834	7,300	498	394	189,942	16.37%
7	0	101,359	13,353	3,184	176	270	118,342	10.20%
8	0	72,287	9,325	1,460	94	838	84,004	7.24%
9	0	57,111	6,070	895	46	11	64,133	5.53%
10	152,127	46,524	3,962	594	51	0	203,258	17.52%
11	90,414	38,631	2,619	422	0	0	132,086	11.39%
12	58,458	30,798	1,896	430	0	0	91,582	7.89%
13	40,161	23,987	1,488	53	0	0	65,689	5.66%
14	27,543	17,686	1,203	0	0	0	46,432	4.00%
15	18,717	12,237	1,451	0	0	0	32,405	2.79%
16	13,469	8,578	1,069	0	0	0	23,116	1.99%
17	9,925	5,919	51	0	0	0	15,895	1.37%
18	7,271	3,962	0	0	0	0	11,233	0.97%
19	5,276	2,745	0	0	0	0	8,021	0.69%
20	3,800	2,077	0	0	0	0	5,877	0.51%
21	2,605	1,480	0	0	0	0	4,085	0.35%
22	1,889	1,344	0	0	0	0	3,233	0.28%
23	1,297	1,670	0	0	0	0	2,967	0.26%
24	853	878	0	0	0	0	1,731	0.15%
25	697	45	0	0	0	0	742	0.06%
>25	2,712	0	0	0	0	0	2,712	0.23%

**Table 4.** Dominant base types and the proportion in genome of *D.anguillare*.

Repeat type	Number of types	Maximum			Minimum		
		Repeat motif	Number	Proportion (%)	Repeat motif	Number	Proportion (%)
Mononucleotide	4	A	191,390	43.77	G	43,065	9.85
Dinucleotide	12	CA	150,240	25.37	GC	604	0.1
Trinucleotide	59	AAT	13,168	14.05	ACG	26	0.03
Tetranucleotide	232	CACG	2,649	8.41	ACCC/ACTT /AGGT/CCAC /CCGA/CGAT /TACG/TGGG	1	0.00
Pentanucleotide	574	TAATG	110	19.16	-	1	0.17
Hexanucleotide	608	CCCTAA	190	7.65	-	1	0.04

## References

[B8338813] Calabrese P., Sainudiin R., Loughin T. M. (2005). Statistical Methods in Molecular Evolution.

[B8338847] Chang C. H., Shao K. T., Lin H. Y., Chiu Y. C., Lee M. Y., Liu S. H., Lin P. L. (2016). DNA barcodes of the native ray-finned fishes in Taiwan. Molecular Ecology Resources.

[B8338859] Chen J. N., López J. A., Lavoué S., Miya M., Chen W. J. (2014). Phylogeny of the Elopomorpha (Teleostei): Evidence from six nuclear and mitochondrial markers. Molecular Phylogenetics and Evolution.

[B8338869] Cui J. Z., Shen X. Y., Yang G. P., Gong Q. L., Gu Q. Q. (2006). The analysis of simple sequence repeats in *Takifugurubripes* genome. Periodical of Ocean University of China.

[B8338889] Duan Y. N., Liu Y., Hu Y. C., Liu C., Song H. M., Wang X. J., Sun J. H., Mu X. D. (2019). Distribution regularity of microsatellites in *Scleropagesformosus* genome. Chinese Agricultural Science Bulletin.

[B8338914] Gao H., Kong J. (2005). Distribution characteristics and biological function of tandem repeat sequences in the genomes of different organisms. Zoological Research.

[B8338923] Hancock J. M. (1996). Simple sequences and the expanding genome. BioEssays.

[B8338932] Harr B., Schlötterer C. (2000). Long microsatellite alleles in drosophila melanogaster have a downward mutation bias and short persistence times, which cause their genome-wide under representation. Genetics.

[B8338950] Hemmer-Hansen J., H&uuml ssy K., Baktoft H., Huwer B., Eero M. (2018). Genetic analyses reveal complex dynamics within a marine fish management area. Evolutionary Applications.

[B9540148] Henkel C. V., Dirks R. P., de Wijze D. L., Minegishi Y., Aoyama J., Jansen H. J., Turner B., Knudsen H., Bundgaard M., Hvam K. L., Boetzer M., Pirovano W., Weltzien F. A., Dufour S., Tsukamoto K., Spaink H. P., van den Thillart G. E.E.J.M. (2012). First draft genome sequence of the Japanese eel, *Anguillajaponica*. Gene.

[B8338941] He X. D., Zheng J. W., He K. Y., Wang B. S. (2015). Comparison of different search tools to find microsatellites sites in unigene sequences of *Salixbabylonica*. Molecular Plant Breeding.

[B8338960] Huang J., Liu L., Yang B., Yang C. Z. (2020). Distribution regularities of microsatellites in the genome of great cormorant (*Phalacrocoraxcarbo*). Chinese Journal of Wildlife.

[B9540171] Jansen H. J., Jansen H. J, Jong-Raadsen S. A., Dufour S., Weltzien F. A., Swinkels W., Koelewijn A., Palstra A. P., Palstra B., Spaink H. P., van den Thillart G. E., Dirks R. P., Henkel C. V. (2017). Rapid de novo assembly of the European eel genome from nanopore sequencing reads. Scientific Reports.

[B8338978] Kajitani R., Toshimoto K., Noguchi H., Toyoda A., Ogura Y., Okuno M., Yabana M., Harada M., Nagayasu E., Maruyama H., Kohara Y., Fujiyama A., Hayashi T., Itoh T. (2014). Efficient de novo assembly of highly heterozygous genomes from whole-genome shotgun short reads. Genome Research.

[B8338997] Kelkar Y. D., Tyekucheva S., Chiaromonte F., Makova K. D. (2008). The genome-wide determinants of human and chimpanzee microsatellite evolution. Genome Research.

[B8339006] Liao Z. Y., Ma Q. Y., Dai X. G., Zhang D. F., Li S. X. (2014). Microsatellite characters in *Juglansregia* L. genome by high throughput sequencing technology. Journal of Northeast Forestry University.

[B8339016] Liu S. D., Xian W. W. (2009). Temporal and spatial patterns of the ichthyoplankton community in the Yangtze Estuary and its adjacent waters. Biodiversity Science.

[B8339025] Maniatis T., Fritsch E. F., Sambrook J. (1982). Molecular cloning: a laboratory manual.

[B8339033] Martin M. (2011). Cutadapt removes adapter sequences from high-throughput sequencing reads. EMBnet Journal.

[B8339042] Messier W., Li S. H., Stewart C. B. (1996). The birth of microsatellites. Nature.

[B8339051] Nelson J. S., Grande T. C., Wilson M. V. H. (2016). Fishes of the World.

[B8339059] Ni S. S., Yang Y., Liu S. F., Zhuang Z. M. (2018). Microsatellite analysis of *Patinopectenyessoensis* using next-generation sequencing method. Progress in Fishery Sciences.

[B9540365] Pavey S. A., Laporte M., Normandeau E., Gaudin J., Letourneau L., Boisvert S., Corbeil J., Audet C., Bernatchez L. (2017). Draft genome of the American eel (*Anguillarostrata*). Molecular Ecology Resources.

[B8339068] Ruan X. H. (2009). Development, characterization and application of microsatellite markers in Turbot. The dissertation of a doctor of pharmaceutical chemistry.

[B8339076] Samadi S., Artiguebielle E., Estoup A., Pointier J. P., Silvain J. F., Heller J., Cariou M. L., Jarne P. (1998). Density and variability of dinucleotide microsatellites in the *parthenogenetic polyploid* snail *Melanoidestuberculata*. Molecular Ecology.

[B8339089] Schlötterer C. (2000). Evolutionary dynamics of microsatellite DNA. Chromosoma.

[B8339098] Schorderet D. F., Gartler S. M. (1992). Analysis of CpG suppression in methylated and nonmethylated species. Proceedings of the National Academy of Sciences.

[B8339107] Sinden R. R., Potaman V. N., Oussatcheva E. A., Pearson C. E., Lyubchenko Y. L., Shlyakhtenko L. S. (2002). Triplet repeat DNA structures and human genetic disease: dynamic mutations from dynamic DNA. Journal of Bioscience.

[B8339118] Song Q., Guo X. G., Chen D. L. (2019). Characterization of microsatellite DNA loci and design of candidate primers to amplify these regions for *Phrynocephalusforsythii* by using 454 GS FLX. Sichuan Journal of Zoology.

[B8339127] Sun B., Bao Y. X., Zhao Q. Y., Zhang L. L., Hu Z. Y. (2009). Methods for obtaining microsatellite loci: A review. Chinese Journal of Ecology.

[B8339137] Tang R. Y., Su M. Y., Yang W. S., Xu J. J., Wang T., Yin S. W. (2022). Analysis of microsatellite distribution characteristics in the channel catfish (*Ictaluruspunctatus*) genome. Progress in Fishery Sciences.

[B8339148] Tautz D., Renz M. (1984). Simple sequences are ubiquitous repetitive components of eukaryotic genomes. Nucleic Acids Research.

[B8339157] Temnykh S., DeClerck G., Lukashova A., McCouch S., Lipovich L., Cartinhour S. (2001). Computational and experimental analysis of microsatellites in rice (*Oryzasativa* L.): Frequency, length variation, transposon associations, and genetic marker potential. Genome Research.

[B8339167] Thiel T., Michalek W., Varshney R., Graner A. (2003). Exploiting EST databases for the development and characterization of gene-derived SSR-markers in barley (*Hordeumvulgare* L.). Theoretical and Applied Genetics.

[B8339176] Wang Y. M., Zhang F. Y., Zhao M., Ma C. Y., Zhang L. Z., Ma L. B. (2019). The complete mitochondrial genome of *Dysommaanguillare* (Anguilliformes, Synaphobranchidae) with phylogenetic consideration. Mitochondrial DNA Part B.

[B8339187] Wang Y. R., Yang W., Ren X. L., Jiang D. N., Deng S. P., Chen H. P., Zhu C. H., Li G. L. (2020). Distribution patterns of microsatellites and development of polymorphic markers from *Scatophagusargus* genome. Journal of Guangdong Ocean University.

[B8339200] Wierdl M., Dominska M., Petes T. D. (1997). Microsatellite instability in yeast: dependence on the length of the microsatellite. Genetics.

[B8339209] Xu J. J., Zheng X., Li J., Yin S. W., Wang T. (2020). Distribution characteristics of whole genome microsatellite of *Pelteobagrusfulvidraco*. Genomics and Applied Biology.

[B8339219] Xu J. J., Zheng X., Zhang X. Y., Wang T., Yin S. W. (2021). Analysis of distribution characteristics of microsatellites in four genomes of puffer fish. Genomics and Applied Biology.

[B8339229] Yang R., Tian S. Q., Gao C. X., Dai L. B., Wang S. C. (2020). Effects of lipid removal on the stable isotopes of *Dysommaanguillaris* in the offshore waters of southern Zhejiang. Journal of Fishery Sciences of China.

[B8339239] Yang T. Y., Huang X. X., Ning Z. J., Gao T. X. (2021). Genome-wide survey reveals the microsatellite characteristics and phylogenetic relationships of *Harpadonnehereus*. Current Issues in Molecular Biology.

[B8339248] Yang W. S., Tang R. Y., Su M. Y., Xu J. J., Wang T., Yin S. W. (2021). Analysis of microsatellite distribution characteristics in the whole genome of *Bagariusyarrelli*. Journal of Nanjing Normal University, Engineering and Technology Edition.

[B8339269] Zhang B., Tang Q. S. (2003). Feeding habits of six species of eels in East China Sea and Yellow Sea. Journal of Fisheries of China.

[B8339278] Zhao R. R., Lu Z. C., Cai S. S., Gao T. X., Xu S. Y. (2021). Whole genome survey and genetic markers development of crocodile flathead *Cociellacrocodilus*. Animal Genetics.

[B8339288] Zhao S. L., Xu H. X., Zhong J. S. (2016). Zhejiang marine ichthyology.

[B8339296] Zhao X., Liu Y. X., Du X. Q., Ma S. Y., Song N., Zhao L. L. (2022). Whole-genome survey analyses provide a new perspective for the evolutionary biology of shimofuri goby, *Tridentigerbifasciatus*. Animals.

